# Phylogenetic relationship of WRKY transcription factors in *Solanum* and potato genes in response to hormonal and biotic stresses

**DOI:** 10.1080/15592324.2025.2491465

**Published:** 2025-04-11

**Authors:** Dequan Jiang, Wenjuan Huang, Jia Liu

**Affiliations:** a Department of Economic Crops Research, Wulanchabu Academy of Agricultural and Forestry Sciences, Wulanchabu, PR China; b Institute of Ecological and Environmental Research, Quzhou Academy of Agricultural and Forestry Sciences, Quzhou, PR China; c College of Agriculture, Inner Mongolia Agricultural University, Hohhot, PR China

**Keywords:** WRKY transcription factors, *Solanum tuberosum*, phylogenetic analysis, abiotic stress, hormone signaling, gene expression

## Abstract

WRKY transcription factors are important regulators of plant responses to environmental stresses and hormone signaling. This study analyzes the WRKY gene family in *Solanum tuberosum* by examining the phylogenetic relationships, expression profiles, and their roles in abiotic stress and hormone responses. Phylogenetic tree was constructed using 322 WRKY genes from four *Solanum* species: *S. tuberosum, S. pennellii, S. pimpinellifolium*, and *S. lycopersicum*. The results revealed conserved and expanded WRKY genes across these species. We then studied the expression of 75 SotuWRKY genes in response to salt, drought, heat stresses, and hormone treatments (IAA, ABA, BABA, GA3, and BAP). Results showed that 19, 25, and 29 genes were regulated under salt, drought, and heat stresses, respectively. Several WRKY genes (e.g. SotuWRKY03 and SotuWRKY24) were also regulated by biotic stresses like *Phytophthora infestans* infection and hormone treatments, indicating their involvement in plant defense mechanisms. A gene co-expression network was constructed based on gene-to-gene correlations, where *SotuWRKY52* was identified as a hub gene, positively regulating six WRKY genes and negatively regulating four. These findings suggest that potato WRKY genes play key roles in regulating stress responses and hormone signaling, potentially enhancing potato resistance to stresses and diseases. This study provides new insights into WRKY transcription factors in *S. tuberosum* and other *Solanum* species.

## Introduction

Transcription factors (TFs) play crucial roles in regulating biological processes such as seed germination, growth, and development by activating or repressing the transcription of target genes.^1^ WRKY is the largest family of transcription factors in plants.^2^ WRKY transcription factors contain one or two conserved WRKY domains (WRKYGQK) and a zinc finger-like motif. The WRKY domain consists of a four-stranded β-sheet,^3–5^ while the zinc finger-like motif forms a zinc-binding pocket, with conserved Cys/His residues located at one end of the β-sheet.^6^ WRKY TFs are classified into three groups (I, II, and III) based on the number of WRKY domains and the type of zinc finger-like motif.^3^ The zinc finger-like motif is further divided into two types: C2H2 (CX4 − 5CX22 − 23HXH) and C2HC (CX7CX23HXC). The members in Group I WRKY TFs contain two WRKY domains and two C2H2 zinc finger-like motifs, while groups II and III members have only one WRKY domain. Group II members have a C2H2 zinc finger motif, whereas the zinc finger-like motif in group III is C2HC, distinguishing them from group II.^7^ Group I is further divided into two subfamilies: I-C and I-N, while group II is divided into five subfamilies (IIa, IIb, IIc, IId, and IIe).^3,4^ The functions of WRKY TFs are to regulate the transcription of target genes by binding to specific cis-elements in the promoter regions, such as the W box (TTGACC/T) and sugar-responsive cis-elements (SURE).^6^ In group I, the two WRKY domains have distinct functions. Most C-terminal WRKY domains in group I mediate specific binding to target DNA sequences, similar to the WRKY domains in groups II and III. However, the function of the N-terminal WRKY domain remains unclear.^3^ SPF1, the first WRKY protein, was identified in sweet potato as early as 1994.^8^ WRKY genes have since been identified in a large number of species with the advancement of sequencing technology. *Chlamydomonas reinhardtii*, a green alga, has a single WRKY gene,^9^ while *Selaginella moellendorffii*, a moss, contains 35 WRKY TFs.^10^ In dicot *Arabidopsis thaliana* and monocot *Oryza sativa* (indica), 72 and 102 WRKY TFs have been identified, respectively.^11,12^ These findings suggest that the number of WRKY family members increases with plant evolution.^2^ However, the number of WRKY TF members does not increase linearly with the genome size of corresponding plant species. For instance, despite having a genome size of 3500 Mb, *Capsicum annuum* only contains 61 CaWRKYs, whereas *Oryza sativa* with a genome size of 390 Mb has 100 WRKYs.^13^

Potato (*S. tuberosum*) is one of the most important staple crops worldwide, particularly in developing countries. Ensuring high yields and improving its tolerance to both abiotic and biotic stresses are of crucial importance for its cultivation. WRKY transcription factors have been identified as key regulators in many biological processes in plants, including responses to biotic and abiotic stresses.^9^ With the completion of the potato genome sequencing,^14^ 82 WRKY transcription factors (TFs) have been identified in potato,^15^ while 79 WRKYs were identified using non-redundant protein sequences.^16^ Additionally, 79 and 84 WRKY genes have been identified in wild potato species.^1^ Although WRKY genes have been identified in many plant species, the functions of only a few members have been elucidated, AtWRKY33 has been reported to regulate tolerance to high-temperature stress in *Arabidopsis thaliana*.^17^ BhWRKY1 has been shown to regulate drought tolerance in *Boea hygrometric*a,^18^ while overexpression of GhWRKY25 reduced drought tolerance in *Nicotiana benthamiana*.^19^ WRKY TFs are not only involved in stress responses but also play a role in regulating hormone signaling pathways.^20^ For instance, OsWRKY53 regulates brassinosteroid signaling and also mediates interactions with other hormonal signaling pathways.^21^ AtWRKY39 is involved in the regulation of both salicylic acid (SA) and methyl jasmonate (MeJA) signaling pathways to help plants cope with high-temperature stress.^22^ These studies suggest that WRKY transcription factors may respond to stresses through the regulation of hormone signaling pathways.

To investigate the regulatory roles of potato WRKY transcription factors in stress responses, 75 WRKY TFs were identified in potato in this study. We performed a comparative phylogenetic analysis based on the amino acid sequences of WRKY genes from various solanaceous plants, including *S. lycopersicum, S. pimpinellifolium, S. pennellii* and *S. tuberosum*. Finally, the expression profiles of the 75 potato WRKY TFs under various biotic, abiotic, and hormone treatments were comprehensively analyzed. The co-expression network was constructed based on the relative expression of 75 *SotuWRKY* genes,^23^ and the characteristics of the potato WRKY family members were extensively discussed.

## Materials and methods

### Genome-wide identification of WRKY TFs in potato

The genomic sequences and coding sequence (CDS sequence) of *S. tuberosum* were downloaded from the publicly available Spud DB Potato Genomics Resource (https://spuddb.uga.edu/pgsc_download.shtml)^24^ and used to construct a local database using BioEdit 7.0 software. The HMM profile of the WRKY domain (Pfam: PF03106) was used as a query to search for candidate WRKY transcription factors (TFs) across the entire potato genome. This search was conducted using the Pfam database (http://pfam-legacy.xfam.org/).^25^ The protein sequences of the putative potato WRKY TFs were then submitted to BLAST for alignment against the potato genome in NCBI (http://blast.ncbi.nlm.nih.gov/Blast.cgi.), with an E-value threshold set to <10^^−5^.

The obtained protein sequences of the WRKY domain were further examined using SMART (http://smart.embl-heidelberg.de/) tools to confirm the presence of the conserved domains.^26^ The theoretical amino acid compositions of the WRKY proteins were analyzed using EXPASY PROTOPARAM (https://web.expasy.org/protparam/).^27^ Chromosomal location for the identified WRKY genes, was collected from the publicly available Spud DB Potato Genomics Resource (https://spuddb.uga.edu/pgsc_download.shtml).^24^ Redundant repeats and incomplete amino acid sequences of conserved domains were removed, along with members having excessively short amino acid sequences.

### Phylogenetic analysis of Solanaceae WRKY genes

The WRKY protein sequences and CDS sequence were downloaded from the publicly available Sol Genomics Network (https://solgenomics.net/.),^28^ including those from *S. lycopersicum*, *S. pimpinellifolium, S. pennellii*, and *S. tuberosum*. The protein sequences of these WRKY transcription factors were subjected to multiple sequence alignment using CLUSTALX 2.0 software with default parameters.^29^ The phylogenetic tree was constructed using MEGA 7.0 software with the Neighbor-Joining method.^30^ Additionally, bootstrap analysis was performed with 2000 iterations and a pairwise gap deletion mode.^31^ The protein sequences of WRKY genes from *S. tuberosum* were compared with those from *S. pimpinellifolium* and *S. pennellii* to identify orthologous gene pairs using OrthoVenn3 (https://orthovenn3.bioinfotoolkits.net/).^32^ The Ka/Ks ratios of the orthologous gene pairs between *S. tuberosum* and *S. pimpinellifolium*/*S. pennellii* were calculated using TBtools with default parameters.^33^

### *Gene expression analysis of* SotuWRKYs

RNA-seq data for *SotuWRKY* genes were retrieved from the publicly available Spud DB Potato Genomics Resource (https://spuddb.uga.edu/index.shtml).^24^ These data was originally published by previous researchers. Fragments Per Kilobase of transcript per Million mapped reads (FPKM) values were directly log₂-transformed and used to investigate the expression patterns of *SotuWRKY* genes in response to various abiotic, biotic and hormonal stresses. Gene expression was classified as upregulated or downregulated according to the criteria described by Ramamoorthy et al.^34^

### *Co-expression analysis of* SotuWRKY *genes*

To assess expression correlations among *SotuWRKY* genes, FPKM values of 75 *SotuWRKY* genes were used to construct a gene co-expression network. RNA-seq data were obtained from the publicly available Spud DB Potato Genomics Resource (https://spuddb.uga.edu/index.shtml).^24^ Co-expression network analysis was performed using the Metware Cloud platform (https://cloud.metware.cn) with default parameters. Expression profiles of *SotuWRKY* genes were used to reveal co-expression relationships under four treatment categories-abiotic stresses (salt, drought, and heat), hormone treatments (IAA, GA₃, BAP, and ABA), chemical inducer treatments (BABA and BTH), and biotic stress (*P. infestans* inoculation).

## Results

### Identification and characterization of WRKY transcription factor genes in potato: distribution, gene lengths, and chromosomal localization

In this study, the HMMER search was used for identifying candidate WRKY proteins in potato. A total of 126 putative candidates were identified; nevertheless, 51 putative candidates were excluded due to redundancy or incomplete domains. The remaining 75 members (*SotuWRKY01-SotuWRKY75*) were retained for further analysis. Detailed information of these WRKY genes, including gene names, sequence IDs, chromosome locations, and gene lengths, was provided in Table 1.

**Table 1.** Characteristics of WRKY proteins from potato (*Solanum tuberosum*).

Gene name	Sequence ID	Chromosome	E-value
SotuWRKY01	PGSC0003DMG400000064	1:72708697–73973628	2.00E–15
SotuWRKY02	PGSC0003DMG400000211	1:73219902–73222113	1.00E–12
SotuWRKY03	PGSC0003DMG400001434	2:45919455–45922325	6.00E–21
SotuWRKY04	PGSC0003DMG400005329	6:1301491–1304767	7.00E–17
SotuWRKY05	PGSC0003DMG400005835	8:5067454–5068479	4.00E–15
SotuWRKY06	PGSC0003DMG400005836	8:5080302–5084470	2.00E–10
SotuWRKY07	PGSC0003DMG400006155	7:44501932–44507039	2.00E–14
SotuWRKY08	PGSC0003DMG400006155	8:40861937–40864121	6.00E–18
SotuWRKY09	PGSC0003DMG400007788	12:1160881–1163790	2.00E–19
SotuWRKY10	PGSC0003DMG400007947	4:67821700–67823360	8.00E–18
SotuWRKY11	PGSC0003DMG400008391	10:53176675–53181319	1.00E–11
SotuWRKY12	PGSC0003DMG400008776	6:34424563–34429400	7.00E–16
SotuWRKY13	PGSC0003DMG400009014	1:62801663–62803440	1.00E–14
SotuWRKY14	PGSC0003DMG400009051	1:62914548–62917002	1.00E–19
SotuWRKY15	PGSC0003DMG400009103	3:44954819–44956436	9.00E–16
SotuWRKY16	PGSC0003DMG400009530	8:2364264–2365922	5.00E–17
SotuWRKY17	PGSC0003DMG400009703	2:30320286–30322046	4.00E–21
SotuWRKY18	PGSC0003DMG400010987	10:54723177–54723177	6.00E–21
SotuWRKY19	PGSC0003DMG400011271	10:751714–755910	3.00E–18
SotuWRKY20	PGSC0003DMG400011457	1:47284761–47286673	3.00E–21
SotuWRKY21	PGSC0003DMG400011633	9:11719670–11722785	4.00E–19
SotuWRKY22	PGSC0003DMG400012160	8:55744934–55747844	2.00E–15
SotuWRKY23	PGSC0003DMG400012317	8:55287077–55289598	2.00E–17
SotuWRKY24	PGSC0003DMG400012318	8:55273301–55275486	2.00E–13
SotuWRKY25	PGSC0003DMG400015015	7:46043497–46046483	9.00E–17
SotuWRKY26	PGSC0003DMG400015076	9:49139319–49141836	2.00E–17
SotuWRKY27	PGSC0003DMG400015104	2:12431776–12435173	9.00E–16
SotuWRKY28	PGSC0003DMG400016441	2:36596829–36599333	2.00E–16
SotuWRKY29	PGSC0003DMG400016769	6:45149200–45151981	4.00E–20
SotuWRKY30	PGSC0003DMG400017349	7:51186199–51189321	9.00E–22
SotuWRKY31	PGSC0003DMG400017990	5:44558173–44671931	2.00E–10
SotuWRKY32	PGSC0003DMG400018061	0:34609383–34609868	2.00E–14
SotuWRKY33	PGSC0003DMG400018081	3:53041664–53045784	5.00E–15
SotuWRKY34	PGSC0003DMG400019706	4:55328944–55333584	1.00E–19
SotuWRKY35	PGSC0003DMG400019824	3:56777004–56780104	1.00E–15
SotuWRKY36	PGSC0003DMG400019884	10:5614014–5617022	4.00E–14
SotuWRKY37	PGSC0003DMG400020206	2:48063218–48065083	1.00E–18
SotuWRKY38	PGSC0003DMG400020432	7:50385331–50389170	7.00E–17
SotuWRKY39	PGSC0003DMG400020608	3:35902713–35905193	9.00E–09
SotuWRKY40	PGSC0003DMG400021895	5:15059048–15061686	2.00E–19
SotuWRKY41	PGSC0003DMG400022063	2:17248582–17250757	5.00E–17
SotuWRKY42	PGSC0003DMG400022143	7:56123389–56129053	2.00E–20
SotuWRKY43	PGSC0003DMG400022290	7:55318791–55323975	3.00E–22
SotuWRKY44	PGSC0003DMG400023196	12:54862748–54865362	7.00E–16
SotuWRKY45	PGSC0003DMG400023360	5:51366941–51372352	5.00E–18
SotuWRKY46	PGSC0003DMG400024961	2:43419448–43420977	8.00E–18
SotuWRKY47	PGSC0003DMG400025481	2:23360208–23362796	6.00E–18
SotuWRKY48	PGSC0003DMG400027208	5:48637085–48638531	1.00E–19
SotuWRKY49	PGSC0003DMG400027582	6:35013158–35015395	4.00E–09
SotuWRKY50	PGSC0003DMG400028335	5:190450–195591	4.00E–22
SotuWRKY51	PGSC0003DMG400028381	5:369472–375911	6.00E–20
SotuWRKY52	PGSC0003DMG400028469	2:31620110–31622139	9.00E–17
SotuWRKY53	PGSC0003DMG400028520	6:50108072–50110187	2.00E–17
SotuWRKY54	PGSC0003DMG400028633	12:49618403–49619840	2.00E–16
SotuWRKY55	PGSC0003DMG400029207	9:18826014–18827752	3.00E–10
SotuWRKY56	PGSC0003DMG400029371	12:58056568–58058005	1.00E–16
SotuWRKY57	PGSC0003DMG400029779	1:81380085–81382117	4.00E–15
SotuWRKY58	PGSC0003DMG400029815	12:9730319–9737930	7.00E–22
SotuWRKY59	PGSC0003DMG400031140	4:61579493–61582536	2.00E–17
SotuWRKY60	PGSC0003DMG400031175	1:69021571–69027216	4.00E–19
SotuWRKY61	PGSC0003DMG400033177	0:34288340–34290610	2.00E–15
SotuWRKY62	PGSC0003DMG400033884	5:44641762–44643847	1.00E–11
SotuWRKY63	PGSC0003DMG400034476	3:45835336–45835857	2.00E–07
SotuWRKY64	PGSC0003DMG400038269	4:47364695–47365888	4.00E–15
SotuWRKY65	PGSC0003DMG400041197	3:44224118–44225093	1.00E–12
SotuWRKY66	PGSC0003DMG400044628	3:44259223–44260629	1.00E–10
SotuWRKY67	PGSC0003DMG400044842	3:45831532–45832311	4.00E–12
SotuWRKY68	PGSC0003DMG401005575	3:25740422–25746456	2.00E–20
SotuWRKY69	PGSC0003DMG401010558	10:3254129–3258564	1.00E–16
SotuWRKY70	PGSC0003DMG401031196	4:54616205–54620206	3.00E–16
SotuWRKY71	PGSC0003DMG401033880	5:44655696–44665602	1.00E–11
SotuWRKY72	PGSC0003DMG402006935	2:25951421–25953871	2.00E–14
SotuWRKY73	PGSC0003DMG402007388	8:40843264–40845641	3.00E–16
SotuWRKY74	PGSC0003DMG402028822	12:6184857–6191565	2.00E–19
SotuWRKY75	PGSC0003DMG402033880	5:44653681–44662748	9.00E–11

Except for two WRKY transcription factors (SotuWRKY32 and SotuWRKY61), the remaining 73 WRKY genes were distributed on chromosomes 1 to 12. Among the 73 genes, chromosome 2 contains the most members, with 10 SotuWRKY genes. In contrast, chromosome 9 has the fewest WRKY gene members, with only 3 members. The members located the remaining chromosomes were ranged from 5 to 9 WRKY genes.

### *Phylogenetic analysis and comparative distribution of WRKY transcription factors in* Solanum *species: insights into gene family expansion and conservation*

**Figure 1.**
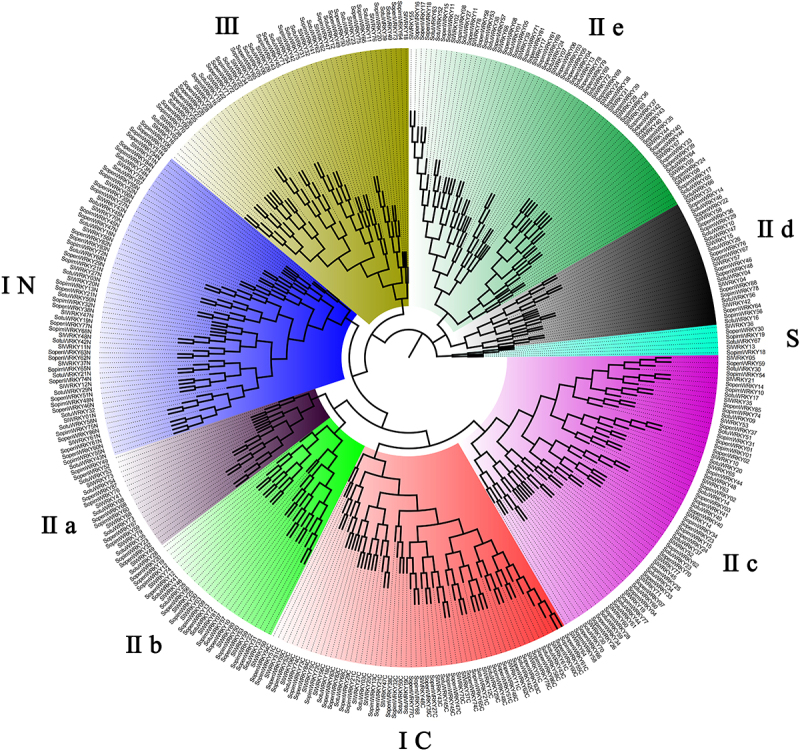
Phylogenetic tree of *WRKY* proteins from *Solanum tuberosum*, *S. pennellii*, *S. pimpinellifolium*, and *S. lycopersicum*, constructed using the Neighbor-Joining method in MEGA 7.0.

To investigate the evolutionary relationships of WRKY transcription factors among *Solanum* species, a phylogenetic tree was constructed using MEGA 7.0 software with the Neighbor-Joining method.^35^ The tree was constructed from 322 WRKY genes, including 75 SotuWRKYs from *S. tuberosum*, 88 SopenWRKYs from *S. pennellii*, 78 SopimWRKYs from *S. pimpinellifolium*, and 81 SlWRKYs from *S. lycopersicum* (Figure 1, Table 2). The *WRKY* genes were categorized into three groups (I, II, and III) based on their phylogenetic relationships and conserved domain structures. Group I consisted of 62 members. With the exception of *SotuWRKY07* and *SotuWRKY32*, which each contained only a single WRKY domain, all other members in this group possessed two WRKY domains. Members of Group I were further classified into the I-N and I-C subgroups, respectively (Figure 1, Table 2). In group I, comparative genomic analysis revealed that genes gain and loss events led to varying numbers of WRKY members across the species: 15 in *S. lycopersicum*, 19 in *S. pennellii*, 14 in *S. pimpinellifolium*, and 14 in *S. tuberosum* (Table 2). The group I displayed more consistent numbers, *S. pimpinellifolium* and *S. tuberosum* each containing 14 members, while *S. lycopersicum* and *S. pennellii* had slight differences.

The sequence of WRKY proteins was clustered into different groups and subgroups, which are labeled with different colors: Group I-C (blue), Group I-N (red), IIa (gray), IIb (green), IIc (purple), IId (black), IIe (dark green), III (brown) and Group S (light green). WRKY genes from each species are denoted as follows: *SotuWRKY* for *S. tuberosum*, *SlWRKY* for *S. lycopersicum*, *SopenWRKY* for *S. pennellii*, and *SopimWRKY* for *S. pimpinellifolium*.

**Table 2.** Group constitution of WRKYs in potato and tomato.

WRKY group		*S. lycopersicum*	*S. pennellii*	*S. pimpinellifolium*	*S. tuberosum*	Total
GroupI		15	19	14	14	62
GroupII		52	54	50	45	201
	IIa	5	5	5	5	20
	IIb	8	8	6	6	28
	IIc	16	16	16	16	64
	IId	6	6	6	10	28
	IIe	17	19	17	8	61
GroupIII		12	14	12	14	53
S		2	1	2	1	6
Total		81	88	78	75	322

Group II exhibited variable membership sizes among species (Table 2). For instance, *S. tuberosum* contained 45 *WRKY* genes in Group II, which were fewer than those found in *S. lycopersicum* (52), *S. pennellii* (54), and *S. pimpinellifolium* (50). Group II was further subdivided into five subgroups (IIa – IIe). Despite overall variation, certain subfamilies within Group II were conserved. For example, five members were consistently identified in Subgroup IIa across all species. In *S. tuberosum*, these included *SotuWRKY35*, *SotuWRKY73*, *SotuWRKY54*, *SotuWRKY08*, and *SotuWRKY53* (Table 3). Similarly, Subgroup IIc contained 16 members in each species. In contrast, subgroup IId exhibited slight variability, with *S. tuberosum* possessing three more members than the other species. Specifically, *SotuWRKY65*, *SotuWRKY66*, and *SotuWRKY64*, along with *SotuWRKY10*, *SotuWRKY46*, *SotuWRKY47*, *SotuWRKY04*, *SotuWRKY26*, *SotuWRKY16*, and *SotuWRKY56* were classified within IId in *S. tuberosum* (Table 3). Group III appeared more conserved, with *S. lycopersicum* and *S. pimpinellifolium* each containing 12 members, while *S. pennellii* and *S. tuberosum* contained 14 (Table 2). A distinct “small group” of six members was identified within this group, including *SlWRKY36*, *SopenWRKY30*, *SopimWRKY19*, *SotuWRKY67*, *SlWRKY13*, and *SopimWRKY18*. Overall, the *S. pennellii* WRKY gene set (*SopenWRKY*) was the largest among the species examined, comprising 88 members-7 more than *S. lycopersicum*, 10 more than *S. pimpinellifolium*, and 13 more than *S. tuberosum*. Despite these differences, the variation in WRKY gene numbers across species within each group was relatively small, reflecting a degree of evolutionary conservation among *Solanum* species.

**Table 3.** Groups of WRKYs in potato.

WRKY group			WRKY name
GroupI		14	SotuWRKY45, SotuWRKY03, SotuWRKY68, SotuWRKY18, SotuWRKY21, SotuWRKY29, SotuWRKY50, SotuWRKY19, SotuWRKY42, SotuWRKY07, SotuWRKY74, SotuWRKY32, SotuWRKY43, SotuWRKY58
GroupII	IIa	5	SotuWRKY35, SotuWRKY73, SotuWRKY54, SotuWRKY08, SotuWRKY53
	IIb	6	SotuWRKY25, SotuWRKY41, SotuWRKY28, SotuWRKY72, SotuWRKY33, SotuWRKY57
	IIc	16	SotuWRKY17, SotuWRKY30, SotuWRKY09, SotuWRKY51, SotuWRKY14, SotuWRKY20, SotuWRKY48, SotuWRKY23, SotuWRKY37, SotuWRKY40, SotuWRKY34, SotuWRKY60, SotuWRKY70, SotuWRKY59, SotuWRKY44, SotuWRKY61
	IId	10	SotuWRKY65, SotuWRKY66, SotuWRKY64, SotuWRKY10, SotuWRKY46, SotuWRKY47, SotuWRKY04, SotuWRKY26, SotuWRKY16, SotuWRKY56
	IIe	8	SotuWRKY13, SotuWRKY69, SotuWRKY01, SotuWRKY24, SotuWRKY38, SotuWRKY27, SotuWRKY52, SotuWRKY63
GroupIII		15	SotuWRKY02, SotuWRKY36, SotuWRKY15, SotuWRKY06, SotuWRKY05, SotuWRKY22, SotuWRKY31, SotuWRKY62, SotuWRKY71, SotuWRKY75, SotuWRKY49, SotuWRKY39, SotuWRKY11, SotuWRKY55, SotuWRKY12
	S	1	SotuWRKY67
Total		75	

To further explore the evolutionary pressures acting on the WRKY gene family in Solanaceae, we calculated Ka/Ks ratios for orthologous WRKY gene pairs between *S. tuberosum* and *S. pimpinellifolium*, as well as between *S. tuberosum* and *S. pennellii* (Tables S1 and S2). The majority of orthologous gene pairs displayed Ka/Ks ratios less than 1 (92.31% and 93.75%, respectively), while a few, such as *SotuWRKY70* and *SotuWRKY63*, showed signs of positive selection, indicating that the *WRKY* gene family in *S. tuberosum* has undergone strong purifying selection, as evidenced by the prevalence of constrained nonsynonymous substitution rates across phylogenetic lineages.

### Expression analysis of WRKY transcription factors in potato: insights into stress response and hormonal regulation

**Figure 2.**
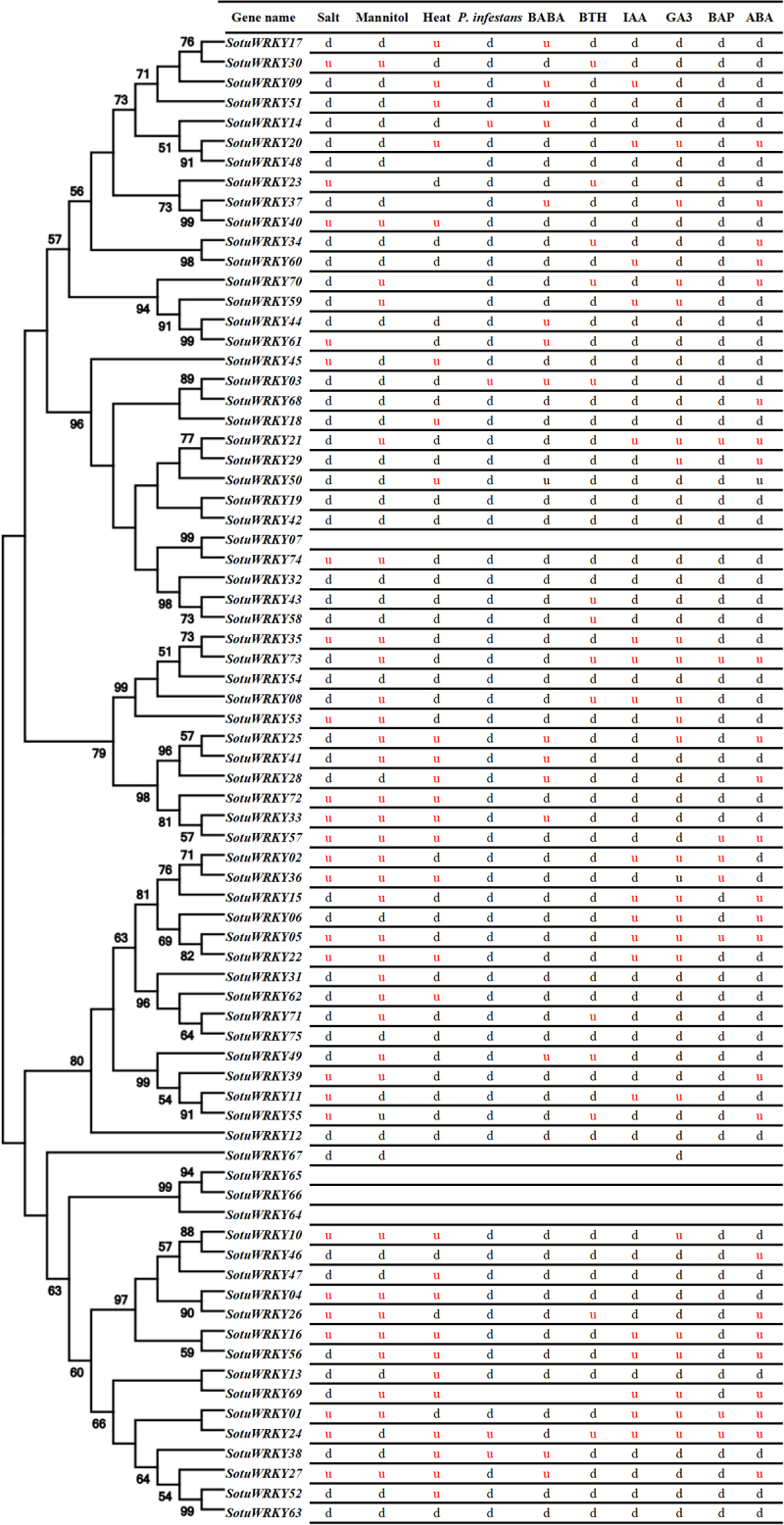
Phylogenetic tree and expression profiles of stress-responsive *WRKY* genes in *solanum tuberosum*.

To further understand the functional roles of SotuWRKY genes in response to stress conditions, expression profiles of 75 SotuWRKY genes were analyzed under abiotic, biotic stresses, and hormone treatments. The abiotic stress treatments included salt (NaCl), drought (mannitol), and heat (35°C), while *Phytophthora infestans* infection was used to evaluate biotic stress. Hormone treatments included BABA, BTH, IAA, GA3, BAP, and ABA. The expression profiles of these genes under various conditions were presented in Figure 2.

A Neighbor-Joining bootstrap consensus tree was constructed for 75 stress-regulated *SotuWRKY* members using the amino acid sequence of a single WRKY domain (the C-terminal domain was used when two domains were present). The graph displays the expression patterns of these genes under various treatments, including abiotic stresses (salinity, drought, and heat), biotic stress (*P. infestans* infection), chemical elicitors (BTH, BABA), and hormone treatments (ABA, IAA, GA₃, and BAP), based on RNA-seq data. Red ‘u’ indicates significant upregulation, while black ‘d’ indicates significant downregulation in response to the corresponding treatment. Treatments are as follows: Salt (NaCl), Drought (Mannitol), Heat (35°C), *P. infestans* (inoculation with *P. infestans*), ABA (abscisic acid), IAA (indole-3-acetic acid), GA₃ (gibberellic acid), BAP (6-benzylaminopurine), BTH (benzothiadiazole), and BABA (β-aminobutyric acid).

A notable finding from the expression analysis was the divergence observed among closely related genes clustered in the phylogenetic tree. While some genes exhibited upregulation (u) or downregulation (d) under specific stress or hormone treatments, others displayed no response (Figure 2), suggesting functional specialization and distinct regulatory roles. In our study, 50 *SotuWRKY* genes were found to be upregulated in response to abiotic stresses. Among them, 23 genes were specifically upregulated by only one of the three abiotic stresses (salinity, drought, or heat), 17 genes responded to two of the stresses, and 10 genes were upregulated under all three stress conditions. These findings underscore the complex regulatory roles of *WRKY* transcription factors in plant responses to abiotic stress. For instance, *SotuWRKY30* was upregulated under both mannitol and salt stress, whereas *SotuWRKY42* was consistently downregulated across multiple treatments, suggesting a stress-specific regulatory mechanism. This variability reflects the intricate network of *WRKY* transcription factor regulation and highlights their diverse functional roles in stress adaptation, including potential involvement in abscisic acid (ABA) signaling pathways.

**Figure 3.**
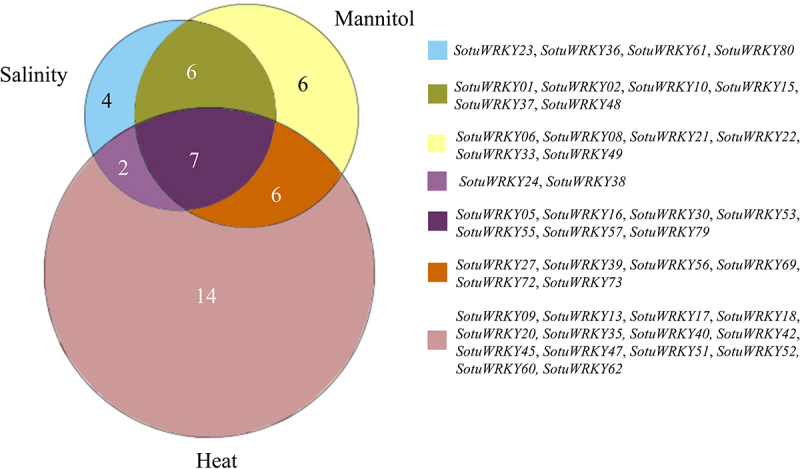
Summary of *SotuWRKY* gene expression under salinity, drought, and heat stress.

Further analysis revealed that 45 *SotuWRKY* genes were significantly regulated by abiotic stresses, including salinity, mannitol, and heat (Figure 3). Among these, 24 genes were specifically responsive to only one stress condition, while 14 genes were regulated by two of the three stresses. This overlap suggests the existence of shared regulatory pathways among different abiotic stress responses. For instance, some genes exhibited combined responsiveness to salinity and mannitol, while others were jointly regulated by heat and one of the other stresses. Notably, seven *SotuWRKY* genes—*SotuWRKY05*, *SotuWRKY16*, *SotuWRKY30*, *SotuWRKY53*, *SotuWRKY55*, *SotuWRKY57*, and *SotuWRKY79*—were co-regulated by all three stress conditions, indicating their potential central roles in mediating cross-stress responses. These genes may function as integrative regulators that coordinate signaling networks, enabling the plant to adapt to a wide range of environmental challenges.

A Venn diagram illustrates the classification of *SotuWRKY* genes that were significantly regulated in response to salinity (NaCl), drought (mannitol), and heat (35°C) treatments based on RNA-seq data. Genes were grouped according to their responsiveness to one or more of the three abiotic stresses. The numbers in each section of the diagram represent the count of genes inducible by the corresponding stress condition(s). A list of the specific *SotuWRKY* genes within each group is provided.

### *Expression profile of* SoutWRKY *genes under* P. infestans *and induced resistance chemical components*

Upon the invasion of *Phytophthora infestans* and the application of resistance-inducing chemical treatments, the transcriptional activity of the 75 SotuWRKY genes exhibited diverse responses, including no change, up-regulation, or down-regulation under various conditions (Figure 2). Specifically, under *P. infestans* infection, 69 *SotuWRKY* genes exhibited altered expression, while six genes showed no significant change. Notably, the majority of genes (65 out of 75, accounting for 86.67%) were downregulated in response to *P. infestans*, whereas only four genes were upregulated. This pattern suggests that most *SotuWRKY* genes may act as negative regulators or are repressed during pathogen infection, reflecting their potential roles in the complex defense response of *S. tuberosum*. These findings suggest a predominantly repressive transcriptional response to late blight in cultivated potato.

The chemical treatments BABA and BTH triggered distinct transcriptional responses among the *SotuWRKY* genes (Figure 2). Under BABA treatment, 15 genes were upregulated, while 6 genes showed no significant change in expression. Similarly, BTH treatment led to the upregulation of 14 genes. In total, 42 *SotuWRKY* genes were collectively induced by both BABA and BTH treatments. Among these, *SotuWRKY03* and *SotuWRKY49* were notably upregulated by both chemical treatments. However, their responses to *P. infestans* infection differed: *SotuWRKY03* was also upregulated following infection, whereas *SotuWRKY49* was downregulated. These findings suggest that while some *SotuWRKY* genes participate in both chemical priming and pathogen response, they may function in partially overlapping but distinct signaling pathways related to pathogen resistance.

Additionally, *SotuWRKY03*, *SotuWRKY14*, and *SotuWRKY38* exhibited up-regulation under both *P. infestans* infection and BABA treatment (Figure 2), highlighting its potential role as a critical regulator in both pathogen-induced and chemically induced resistance pathways. Similarly, *SotuWRKY24* showed dual up-regulation in response to *P. infestans* infection and BTH treatment, further emphasizing its role in integrating pathogen and chemical resistance signals, while *SotuWRKY03* was up-regulated by both *P. infestans*, BABA, and BTH.

### *The expression profile of* SotuWRKY *transcription factors under hormone treatment*

**Figure 4.**
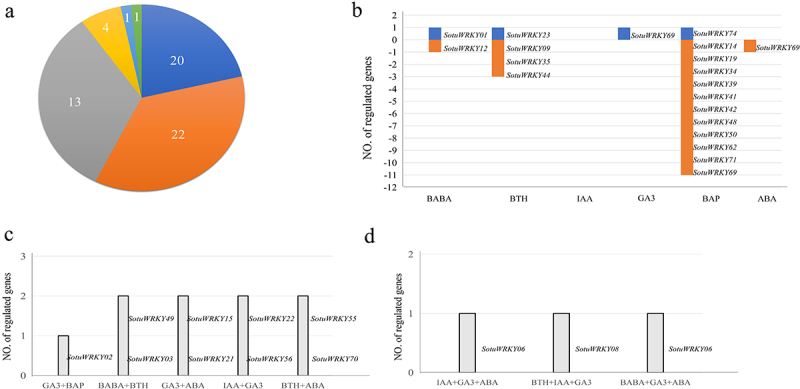
Summary of *SotuWRKY* gene expression under various hormone treatments.

Plant hormones play a crucial role in regulating plant growth and development. To investigate the molecular mechanisms by which *SotuWRKY* genes respond to hormonal signals, the expression profiles of 61 SotuWRKY genes were analyzed under six hormone treatments: IAA, ABA, BABA, BTH, GA3, and BAP (Figure 4a). The results revealed a diverse and specific pattern of gene expression under different hormonal conditions. Among the 61 *SotuWRKY* genes responsive to hormone treatments, 20 were regulated by a single hormone, 22 by two hormones, 13 by three hormones, and 4 by four hormone treatments. Additionally, one gene was regulated by five hormones, and another was influenced by six. Notably, *SotuWRKY01*, *SotuWRKY05*, *SotuWRKY21*, *SotuWRKY24*, and *SotuWRKY73* were co-regulated by IAA, GA₃, BAP, and ABA (Figure 2), suggesting their involvement in multiple hormone signaling pathways. In contrast, genes that responded exclusively to a single hormone treatment – such as *SotuWRKY03*, *SotuWRKY04*, and *SotuWRKY11*—may play more specialized roles in specific hormone-mediated signaling processes. This diversity in hormone responsiveness highlights the functional specialization and complexity of *SotuWRKY* gene regulation within plant hormone signaling networks.

(a) A pie chart illustrating the number of *SotuWRKY* genes regulated by one to six hormone treatments. Genes regulated by a single hormone are shaded in blue, two hormones in orange, three hormones in gray, four hormones in yellow, five hormones in light blue, and six hormones in green. (b) Bar graph showing *SotuWRKY* genes regulated by only one hormone. Blue bars represent the number of genes upregulated by each hormone, with corresponding gene names listed to the right of each column. Orange bars indicate the number of downregulated genes, also with gene names listed accordingly. (c, d) Lists of genes regulated by two (c) and three (d) hormone treatments, respectively. Shaded bars represent the number of genes upregulated in response to specific combinations of hormone treatments. Corresponding gene names are shown to the right of each column.

Among the 20 genes that responded to a single hormone treatment (Figure 4b), four genes (*SotuWRKY01*, *SotuWRKY23*, *SotuWRKY69*, and *SotuWRKY74*) were up-regulated, while the remaining genes were down-regulated. Notably, no gene was exclusively regulated by IAA, suggesting a more complex interaction with IAA signaling. However, 12 genes showed significant responsiveness to BAP, indicating a strong regulatory role of this hormone in *SotuWRKY* gene expression. This finding underscores the potential dominance of BAP in modulating the transcriptional activity of specific *SotuWRKY* genes.

For genes regulated by two hormone treatments (Figure 4c), their expression patterns displayed consistent up-regulation in response to both hormones. For instance, *SotuWRKY02* responded to both GA3 and BAP, highlighting its potential dual role in hormone-mediated pathways. Such genes may act as key mediators of crosstalk between distinct hormonal signals. In contrast, genes regulated by three hormones exhibited more complex and varied expression patterns (Figure 4d), indicating their involvement in integrating multiple hormonal signals. One notable example is *SotuWRKY06*, which was up-regulated by IAA, GA3, and ABA. This unique response pattern suggests that *SotuWRKY06* may serve as a central hub in the hormonal regulatory network. *SotuWRKY08* demonstrated up-regulation in response to BTH, IAA, and GA3, further emphasizing its potential role in coordinating multiple hormonal pathways.

### WRKY genes regulated by the combination of abiotic stress and hormone treatments

**Figure 5.**
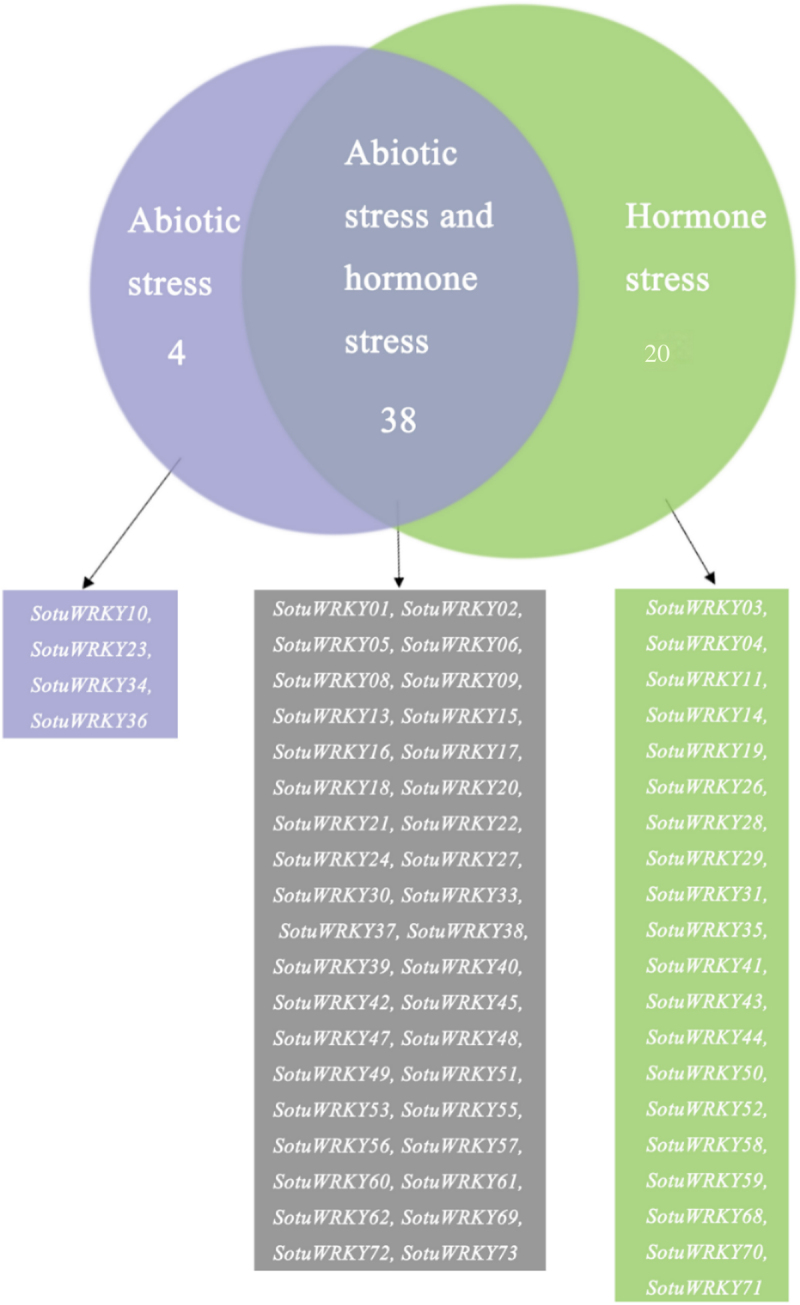
Summary of expression analyses of 62 stress-responsive *SotuWRKY* genes under both abiotic and hormone treatments. A venn diagram illustrates the classification of *SotuWRKY* genes that were significantly regulated by abiotic stresses (salt, drought, and heat) and/or various hormone treatments (ABA, IAA, GA₃, and BAP) based on RNA-seq data. Genes were grouped into three categories: those regulated exclusively by abiotic stress (shaded in purple), exclusively by hormone treatments (shaded in green), and by both abiotic and hormone treatments (shaded in gray). A list of the *SotuWRKY* genes in each group is provided below the diagram.

Based on the data presented in Figure 5, 24 WRKY genes were found to be exclusively regulated either by abiotic stress or hormone treatments, while 38 genes were influenced by both types of treatments. This finding highlights the diversity in the roles played by *SotuWRKY* genes, as some are specialized for abiotic stress responses or hormone signaling, whereas others act as integrators of these pathways. Specifically, four genes (*SotuWRKY10*, *SotuWRKY23*, *SotuWRKY34*, and *SotuWRKY36*) were solely responsive to abiotic stress conditions, while 20 genes, including *SotuWRKY03*, *SotuWRKY04*, and *SotuWRKY11*, were exclusively influenced by hormone treatments. The majority of the genes, however, were regulated by both stress types, suggesting a critical role in coordinating the interaction between abiotic stress responses and hormone-mediated signal.

### Co-expression network analysis of SotuWRKYs

**Figure 6.**
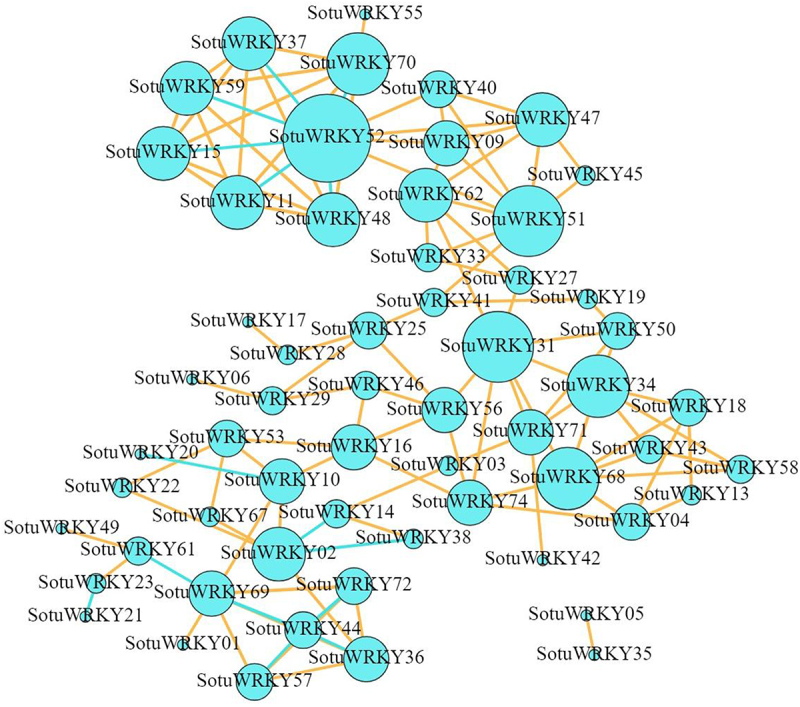
Co-expression network of *SotuWRKY* genes.

A co-expression network of *SotuWRKY* genes was constructed using expression profiles derived from four treatment categories: abiotic stresses (salt, drought, and heat), hormone treatments (IAA, GA₃, BAP, and ABA), chemical elicitors (BABA and BTH), and biotic stress (*P. infestans* inoculation) (Figure 6). The 75 *SotuWRKY* genes were grouped into two distinct clusters. The major cluster comprised 73 co-expressed genes, while the minor cluster contained only two members: *SotuWRKY05* and *SotuWRKY35*. Within the primary co-expression network, *SotuWRKY52* emerged as a central hub gene, showing positive regulatory interactions with 10 genes and negative interactions with 6 genes (*SotuWRKY37*, *SotuWRKY59*, *SotuWRKY15*, *SotuWRKY11*, *SotuWRKY48*, and *SotuWRKY70*). Additionally, *SotuWRKY51* and *SotuWRKY31* each displayed positive regulatory associations with 8 other genes. Overall, only 17 genes were involved in negative regulatory interactions, while the majority exhibited mutually positive co-regulation. These results suggest that most *SotuWRKY* genes participate in positive feedback relationships within the network, indicating potential cooperative functions. The high degree of co-expression and mutual regulation implies that *SotuWRKY* genes may act synergistically in regulating responses to biotic, abiotic, and hormonal stresses, likely contributing to shared or overlapping biological processes.

Each blue node represents a *SotuWRKY* gene. The size of the node reflects the number of co-expressed gene connections, with larger nodes indicating higher connectivity. Solid lines represent co-expression relationships: orange lines denote positive correlations, while blue lines indicate negative correlations between genes.

## Discussion

### Annotation of WRKY genes in potato genome

Three studies have identified WRKY transcription factors in potato, with 79 members in cultivated potato,^16^ 82 members in *S. commersonii*,^15^ and 79 and 84 members in *S. chacoense*, two wild potato lines.^1^ In the present study, we removed genes with incomplete domains and redundancies, ultimately identifying 75 *SotuWRKY* genes in potato. Previous researches showed that tandem duplication gene pairs and segmental duplication gene pairs found from *StWRKY* family genes.^16^ The comprehensive expression profiles of all 75 potato SotuWRKY genes were analyzed in detail.

### Phylogenetic grouping and gene family expansion

This study provided valuable insights into the evolutionary patterns and functional diversification of WRKY transcription factors (TFs) in four *Solanum* species: *S. tuberosum, S. pennellii, S. pimpinellifolium*, and *S. lycopersicum*. Through the analysis of 322 *WRKY* genes, we identified both conserved and species-specific patterns in gene family distribution, reflecting potential adaptations to distinct environmental conditions. The phylogenetic analysis grouped the WRKY genes into several main clusters, with notable differences in gene numbers between species. *S. tuberosum* had the fewest WRKY genes in group I (14 genes), compared to other species like *S. pennellii* (19 genes). This suggests that the potato genome may have undergone gene loss or reduced expansion in the WRKY gene family. On the other hand, wild species like *S. pennellii* and *S. pimpinellifolium* showed greater WRKY gene expansion, which could be a result of their adaptation to diverse environmental stresses, such as drought or pathogens.

While the WRKY gene family showed some species-specific differences, there were also highly conserved subfamilies. For example, subfamilies IIa and IIc contained the same number of WRKY genes across all four species (five genes in IIa and sixteen in IIc). This indicates that these WRKY genes likely play fundamental roles in basic plant functions, such as growth and stress responses, and are conserved across species. In group III, the WRKY genes were more conserved across species, with only a small variation in gene numbers between the species. The presence of a “small group” within group III, containing six specific WRKY genes, suggests that these genes may play specialized roles in stress response, such as pathogen defense. This group might be crucial for regulating core stress-related pathways in the plants.

*S. pennellii*, an important species in the evolutionary lineage between potato and tomato, is classified under the Solanum genus in modern taxonomy but was previously grouped under the potato subgenus, and was also analyzed for comparison. The differences in the number of WRKY genes between cultivated potato (*S. tuberosum*) and wild species suggest that domestication might have led to a more compact WRKY gene family in cultivated potatoes. In contrast, wild species like *S. pennellii* have retained a larger WRKY gene pool, potentially providing them with more tools for adapting to environmental stress.

In the co-expression network, genes with similar functions or involved in the same regulatory pathways tend to cluster within the same module, following the “guilt-by-association” principle.^36^ This feature enables the functional prediction of uncharacterized genes based on their co-expression with well-annotated genes.^37^ Notably, 73 out of the 75 *SotuWRKY* genes were integrated into a single co-expression network, indicating potential functional similarity or synergistic interactions among them. Only *SotuWRKY05* and *SotuWRKY35* were excluded from this main network, suggesting they may function independently or participate in distinct regulatory pathways.

### Molecular characterization and stress response profiling of SotuWRKY transcription factors in potato: insights into abiotic and biotic stress regulation

Environmental stressors, exacerbated by climate change, are major barriers to agricultural sustainability. Enhancing the stress resilience of crops is a critical strategy to ensure global food security.^38^ In this study, we examined the expression of 75 potato SotuWRKY genes under different biotic, abiotic, and hormonal stress conditions. Our results showed that 25 genes were upregulated by salinity, 33 by drought and 28 by heat treatment (Figure 2). For example, *SotuWRKY10* and *SotuWRKY36* could be key regulators in abiotic stress-specific networks, given their unique responsiveness.

WRKY transcription factors have been shown to play crucial roles in the regulation of plant responses to various stresses. In Arabidopsis, *WRKY48* acts as a negative regulator of the plant’s transcriptomic response to combined high light and heat stress.^39^ In rice, WRKY genes are involved in responses to drought, cold, heat, and salinity stresses. For instance, *OsWRKY7* coordinates alternative translation and protein stability, influencing plant growth and basal defense mechanisms.^40^ In *Capsicum annuum, LrWRKY3/27* regulates anthocyanin production and contributes to drought response.^41^ It is well established that abiotic stress-regulated genes can act in an ABA-dependent or ABA-independent manner.^42^ For example, *TaWRKY29* positively regulated the ABA signaling pathway and drought response by binding to the *TaABI55* promoter in *Triticum aestivum*.^43^ Similarly, *ZmWRKY79* in *Zea mays* positively regulates drought tolerance by promoting ABA biosynthesis.^44^ The genes exclusively regulated by abiotic stress likely function in direct response to environmental factors such as drought, salinity, or heat. These genes might regulate protective mechanisms that help plants cope with challenging conditions by adjusting physiological and biochemical pathways.

Previous studies on wild potato have shown that *StWRKY* gene expression remains largely unaffected by *P. infestans* infection.^1^ This discrepancy highlights significant differences in the defense mechanisms between cultivated and wild potato varieties in response to late blight. In our study, several *SotuWRKY* genes, such as *SotuWRKY14* and *SotuWRKY38*, were responsive to both *P. infestans* infection and chemical inducers BABA and BTH. Notably, *SotuWRKY24* was regulated by both *P. infestans* and BTH, while *SotuWRKY03* was induced by *P. infestans*, BABA, and BTH. BABA-induced resistance (BABA-IR) has emerged as a well-established model for studying defense priming mechanisms, as it enhances a plant’s capacity to mount faster and stronger responses upon subsequent stress exposure.^45^ Meanwhile, BTH, a widely used commercial resistance inducer, has been extensively applied in agricultural systems to boost plant defense against various pathogens.^46^ The responsiveness of these *SotuWRKY* genes to both pathogen infection and chemical elicitors suggests their potential roles in bridging innate immune signaling and priming pathways. Functional evidence from other species supports the involvement of WRKYs in resistance responses. For example, *NaWRKY70* in *Nicotiana attenuata* regulates capsidiol biosynthesis, a key antimicrobial compound, and its silencing results in reduced ABA production and compromised pathogen defense.^30^ In *S. tuberosum*, *StWRKY8* is involved in the biosynthesis of benzylisoquinoline alkaloids (BIAs), which possess antimicrobial activity and contribute to cell wall reinforcement, thereby limiting pathogen spread.^47^ The dual responsiveness of certain *SotuWRKY* genes to both chemical inducers and pathogen attacks underscores their roles as molecular hubs in plant resistance networks. Further investigation is warranted to elucidate the precise regulatory mechanisms linking these WRKY genes to signaling pathways involved in pathogen defense and resistance priming.

### WRKY transcription factors in stress responses and Hormonal Regulation: insights into plant resilience and Growth Regulation

In this study, we analyzed the expression profiles of *SotuWRKY* genes under various hormone treatments and identified a substantial group of 38 genes that were responsive to both abiotic stresses and hormonal stimuli. Notably, genes such as *SotuWRKY01*, *SotuWRKY06*, *SotuWRKY17*, and *SotuWRKY33* (Figure 5) emerged as key candidates potentially involved in the crosstalk between abiotic stress responses and hormone-mediated signaling pathways. These dual-responsive genes may play pivotal roles in regulating not only stress responses but also fundamental developmental processes such as growth, senescence, and organogenesis.

Their hormone-specific regulation suggests a specialized function within hormone signaling networks, enabling the plant to adapt to environmental fluctuations through hormonal modulation. This dual regulation implies that these *WRKY* genes serve as integrative nodes, linking external environmental signals with internal hormonal cues to coordinate appropriate physiological responses. Such integration is critical for optimizing resource allocation, allowing plants to balance growth and stress tolerance under adverse conditions.

WRKY transcription factors are key regulators of secondary metabolites in plants, influencing their response to stress by mediating hormone synthesis and breakdown.^48^ This regulation is central to processes such as seed germination, growth, flowering, and defense against pathogens and pests.^10,38^ WRKYs thus represent an important mechanism for plants to adapt to their environment, balancing growth and development with the need to respond to stress.^2,48^ This suggests that WRKYs play a crucial role in plant adaptations to a stationary lifestyle, helping coordinate stress responses with growth and development.^10^ WRKYs act as regulatory hubs, maintaining a balance between stress responses and plant growth, especially during the vegetative stage. By influencing photomorphogenesis, shoot branching, plant height, and stem development, WRKYs modulate the progression of plant maturation.^48^

To further investigate the role of WRKY genes in hormone regulation and stress responses, we plan to silence one of these key WRKY genes. We will employ various techniques, including electrophoretic mobility shift assays, chromatin immunoprecipitation, transient overexpression, and virus-induced gene silencing, to explore the intricate relationships between WRKY genes and hormone signaling pathways.

## Conclusion

This study demonstrates the diverse roles of SotuWRKY transcription factors in potato under various stress and hormonal treatments. Among the 75 analyzed genes, some responded specifically to a single hormone, while others were regulated by multiple hormones, highlighting their functional specialization. Key genes like *SotuWRKY06* and *SotuWRKY02* showed the ability to integrate signals from multiple hormonal pathways, suggesting their central roles in regulatory networks. BAP was identified as a critical hormone influencing the expression of several SotuWRKY genes. In addition, the combination of abiotic stress (salt, drought, and heat) with hormonal treatments revealed distinct gene responses. Some genes, such as *SotuWRKY10* and *SotuWRKY36*, were exclusively responsive to abiotic stress, while others, like *SotuWRKY03* and *SotuWRKY11*, were regulated only by hormones. Notably, 38 genes were responsive to both stress and hormones, indicating their role in coordinating environmental and hormonal signals. These findings provide a foundation for understanding the regulatory functions of *SotuWRKY* genes and their potential application in improving potato stress tolerance and growth under challenging conditions.

## Supplementary Material

Supplemental_Table_S1 clean.doc

Supplemental_Table_S2 clean.doc
