# Supplementary material for: Phylogenetic relationship of WRKY transcription factors in Solanum and potato genes in response to hormonal and biotic stresses
Source: Plant Signal Behav. 2025 Apr 11;20(1):2491465. doi: 10.1080/15592324.2025.2491465 (PMC12716037; doi:10.1080/15592324.2025.2491465)
Supplement: Supplemental_Table_S1 clean.doc [file KPSB_A_2491465_SM3208.doc]

Supplemental Table S1 Ka, Ks and Ka/Ks value for WRKYs orthology gene pairs of potato and wild tomato

| Gene name | Chr | Gene name | Chr | Ka | Ks | Ka/Ks |
| --- | --- | --- | --- | --- | --- | --- |
| SotuWRKY14 | 1 | SopenWRKY03 | 1 | 0.06 | 0.12 | 0.49 |
| SotuWRKY02 | 1 | SopenWRKY09 | 1 | 0.02 | 0.12 | 0.18 |
| SotuWRKY57 | 1 | SopenWRKY10 | 1 | 0.02 | 0.07 | 0.23 |
| SotuWRKY27 | 2 | SopenWRKY11 | 2 | 0.04 | 0.18 | 0.25 |
| SotuWRKY41 | 2 | SopenWRKY12 | 2 | 0.03 | 0.14 | 0.18 |
| SotuWRKY72 | 2 | SopenWRKY13 | 2 | 0.03 | 0.11 | 0.26 |
| SotuWRKY33 | 3 | SopenWRKY31 | 3 | 0.04 | 0.17 | 0.22 |
| SotuWRKY34 | 4 | SopenWRKY33 | 4 | 0.01 | 0.07 | 0.21 |
| SotuWRKY70 | 4 | SopenWRKY34 | 4 | 0.11 | 0.11 | 1.04 |
| SotuWRKY51 | 5 | SopenWRKY37 | 5 | 0.01 | 0.05 | 0.27 |
| SotuWRKY29 | 6 | SopenWRKY51 | 6 | 0.01 | 0.08 | 0.16 |
| SotuWRKY25 | 7 | SopenWRKY56 | 7 | 0.04 | 0.14 | 0.29 |
| SotuWRKY09 | 12 | SopenWRKY85 | 12 | 0.04 | 0.15 | 0.30 |
| SotuWRKY14 | 1 | SopimWRKY02 | 1 | 1.45 | 1.96 | 0.74 |
| SotuWRKY13 | 1 | SopimWRKY03 | 1 | 1.45 | 1.93 | 0.75 |
| SotuWRKY02 | 1 | SopimWRKY06 | 1 | 1.74 | 2.36 | 0.74 |
| SotuWRKY57 | 1 | SopimWRKY07 | 1 | 0.01 | 0.07 | 0.20 |
| SotuWRKY27 | 2 | SopimWRKY08 | 2 | 0.05 | 0.17 | 0.31 |
| SotuWRKY72 | 2 | SopimWRKY09 | 2 | 0.04 | 0.12 | 0.29 |
| SotuWRKY15 | 3 | SopimWRKY16 | 3 | 0.04 | 0.15 | 0.25 |
| SotuWRKY65 | 3 | SopimWRKY17 | 3 | 0.13 | 0.25 | 0.53 |
| SotuWRKY63 | 3 | SopimWRKY18 | 3 | 0.18 | 0.14 | 1.28 |
| SotuWRKY33 | 3 | SopimWRKY22 | 3 | 0.03 | 0.16 | 0.21 |
| SotuWRKY34 | 4 | SopimWRKY25 | 4 | 0.02 | 0.06 | 0.35 |
| SotuWRKY70 | 4 | SopimWRKY26 | 4 | 0.12 | 0.12 | 0.97 |
| SotuWRKY10 | 4 | SopimWRKY29 | 4 | 0.01 | 0.12 | 0.11 |
| SotuWRKY75 | 5 | SopimWRKY42 | 5 | 0.05 | 0.09 | 0.58 |
| SotuWRKY04 | 6 | SopimWRKY46 | 6 | 0.01 | 0.13 | 0.05 |
| SotuWRKY09 | 12 | SopimWRKY74 | 12 | 0.05 | 0.13 | 0.39 |
