# Supplementary material for: Phylogenetic relationship of WRKY transcription factors in Solanum and potato genes in response to hormonal and biotic stresses
Source: Plant Signal Behav. 2025 Apr 11;20(1):2491465. doi: 10.1080/15592324.2025.2491465 (PMC12716037; doi:10.1080/15592324.2025.2491465)
Supplement: Supplemental_Table_S2 clean.doc [file KPSB_A_2491465_SM3207.doc]

Supplemental Table S2 Statistics of WRKY orthology gene pairs of potato with wild tomato gene

| Species | Total | Ka/Ks>1 | % | Ka/Ks | % |
| --- | --- | --- | --- | --- | --- |
| *S. tuberosum-S. pennellii* | 13 | 1 | 7.69 | 12 | 92.31 |
| *S. tuberosum-S. pimpinellifolium* | 16 | 1 | 6.25 | 15 | 93.75 |
